# Structure and Dynamics of Antigenic Peptides in Complex with TAP

**DOI:** 10.3389/fimmu.2017.00010

**Published:** 2017-01-30

**Authors:** Elisa Lehnert, Robert Tampé

**Affiliations:** ^1^Institute of Biochemistry, Biocenter, Goethe University Frankfurt, Frankfurt, Germany

**Keywords:** ABC transporter, antigen processing, ligand binding, membrane proteins, peptide-loading complex, substrate-binding site

## Abstract

The transporter associated with antigen processing (TAP) selectively translocates antigenic peptides into the endoplasmic reticulum. Loading onto major histocompatibility complex class I molecules and proofreading of these bound epitopes are orchestrated within the macromolecular peptide-loading complex, which assembles on TAP. This heterodimeric ABC-binding cassette (ABC) transport complex is therefore a major component in the adaptive immune response against virally or malignantly transformed cells. Its pivotal role predestines TAP as a target for infectious diseases and malignant disorders. The development of therapies or drugs therefore requires a detailed comprehension of structure and function of this ABC transporter, but our knowledge about various aspects is still insufficient. This review highlights recent achievements on the structure and dynamics of antigenic peptides in complex with TAP. Understanding the binding mode of antigenic peptides in the TAP complex will crucially impact rational design of inhibitors, drug development, or vaccination strategies.

## Introduction

Our human body is continually threatened by billions of potential pathogens, e.g., bacteria, viruses, fungi, and parasites. Thus, a multilayered defense has evolved to protect vertebrates from these pathogens by sophisticated mechanisms. Physical and chemical barriers, such as skin or gastric juice, are the primary, non-specific protective shields preventing the pathogens from entering the host organism. Pathogens able to pass this first barrier are combated by the innate immune system as the secondary protective shield reacting with an immediate, pathogen-oriented response mediated by immune cells, such as macrophages, granulocytes, and natural killer cells, or the plasma protein cascade of the complement system. As third layer of defense, the adaptive immune system recognizes antigens and mounts an immunological memory. Adaptive immunity acts *via* a humoral and cellular response. The humoral, antibody-mediated response depends on the antigen/pathogen recognition by B-lymphocytes within the lymph or blood. However, the cellular path of adaptive immunity utilizes T-lymphocytes recognizing antigenic peptides presented by major histocompatibility complexes (MHC). This pathway has regulatory and cytotoxic functions (1).

Antigen presentation can be subdivided into MHC class I and MHC class II dependent pathways. Antigenic peptides derived from exogenous antigens are loaded in lysosomal-like compartments on MHC II molecules and are finally presented to CD4^+^ T helper lymphocytes (2, 3). Endogenous antigens are degraded *via* the ubiquitin/proteasome and other proteolytic pathways. Degradation products can be translocated into the lumen of the endoplasmic reticulum (ER) by the transporter associated with antigen processing (TAP). A peptide-loading complex (PLC), composed of TAP1 and TAP2, the two ER chaperones, tapasin, and calreticulin, the oxidoreductase ERp57 together with MHC I heavy chain and β_2_-microglobulin, is essential for efficient loading of antigenic peptides onto MHC I molecules. After epitope proofreading and quality control within the PLC, kinetically stable peptide–MHC complexes are released to shuttle their antigenic cargo *via* the secretory pathway to the plasma membrane. At the cell surface, MHC I molecules present their antigenic peptides to CD8^+^ cytotoxic T-lymphocytes, which eventually induce the elimination of virally or malignantly transformed cells (2, 3). Cross-presentation is a subtype of MHC I-dependent antigen presentation but mediated by efficient uptake and processing of exogenous antigens. Two main pathways for cross-presentation are proposed. However, the exact mechanistic details are still unclear. While the cytosolic pathway is proteasome- and TAP-dependent, the vacuolar pathway depends on neither the proteasome nor TAP (4).

The fundamental role of the transport complex TAP within the adaptive immunity predestinates TAP as a target for infectious diseases and malignant disorders, such as bare lymphocyte syndrome type I and cancer. Detailed knowledge about the TAP structure and transport mechanism is thus of capital importance for the development of therapies or drugs against such diseases, but numerous aspects are insufficiently identified to date. This review focuses on the structure and dynamics of antigenic peptides bound to TAP, shedding light on recent efforts to determine the structure of a bound substrate and to localize its respective binding site by biophysical and theoretical methods, such as electron paramagnetic resonance (EPR), nuclear magnetic resonance (NMR), and molecular docking experiments.

## Structural Arrangement of the Human TAP Complex

**Figure 1:**
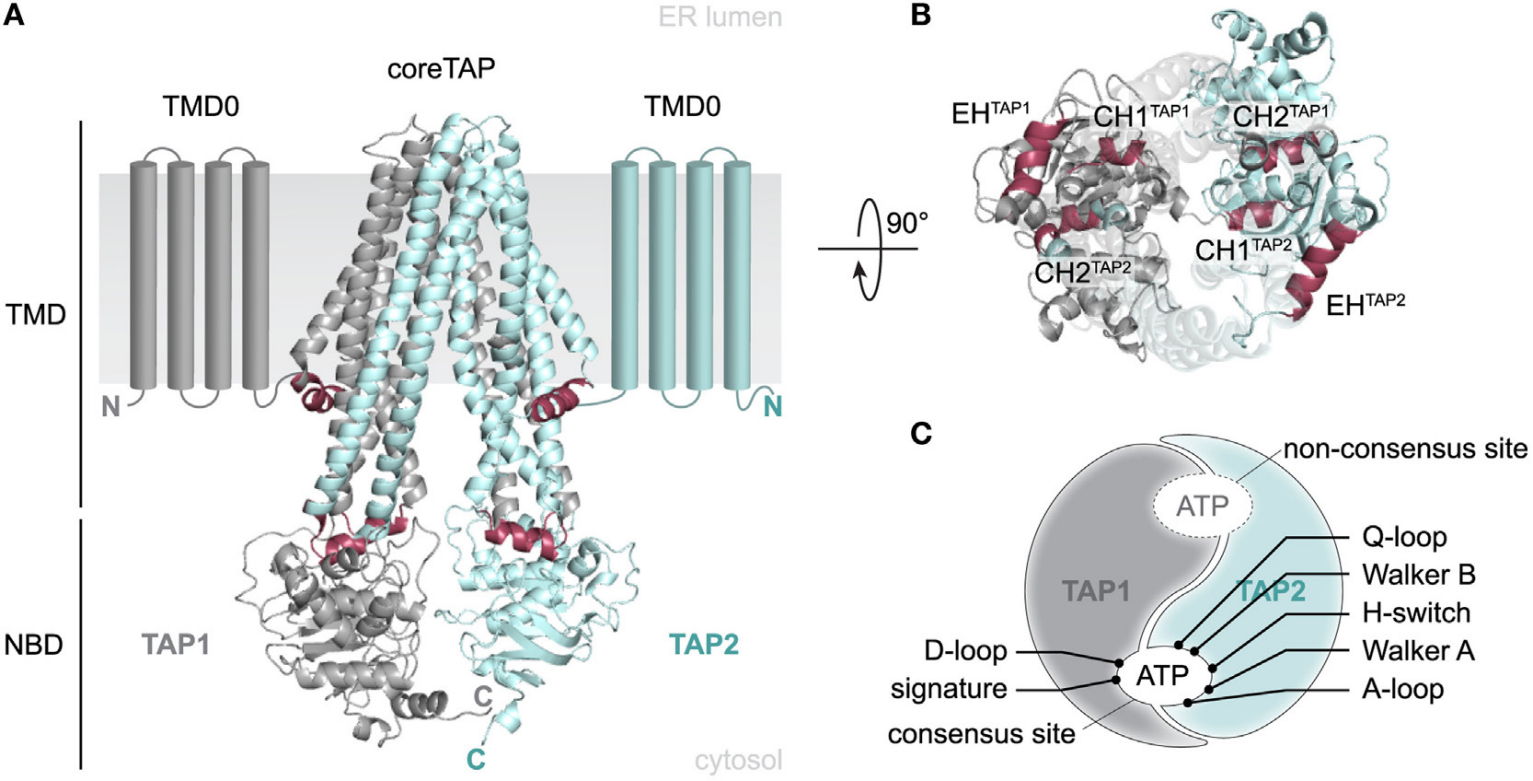
**Structural organization of the antigen translocation complex transporter associated with antigen processing (TAP)**. **(A)** 3D homology model of the human TAP complex based on the TAP-related heterodimeric ABC-binding cassette transporter TmrAB in the inward-facing conformation (13, 14). The heterodimeric translocation machinery (TAP1 and TAP2) consists of a 2 × 6 TMHs core domain, two additional N-terminal four-transmembrane helix bundles (TMD0, schematically shown), and two nucleotide-binding domains (NBDs). Core transmembrane domains (TMDs) and TMD0s are connected *via* elbow helices (EHs). Two NBDs facilitate ATP binding and hydrolysis. **(B)** Top view from the ER lumen along the TMD–NBD interface. Coupling helix CH1 mediates inter-domain cross talk between NBDs and TMDs *in cis* and *in trans*, whereas CH2 interacts *in trans*. **(C)** Schematic view of the asymmetric NBDs of the TAP complex. NBDs are tightly packed in the outward-facing conformation and thus form two ATP-binding sites. The presence of non-equivalent, consensus and non-consensus ATPase sites is based on aberrant amino acid residues within the conserved sequence motifs. TAP1: gray, TAP2: blue, CH/EH: raspberry.

TAP1 and TAP2 are members of the ABC-binding cassette (ABC) subfamily B (ABCB2 and ABCB3) and found in all nucleated cells of jawed vertebrates. TAP is predominantly located in the ER and *cis*-Golgi, although an ER-targeting or ER-retention signal has not been specified to date (5). A heterodimeric TAP complex is essential and sufficient for peptide binding and translocation, whereas TAP1 or TAP2 homodimers are non-functional (6, 7). TAP consists of two transmembrane domains (TMDs) harboring the substrate-binding site and two nucleotide-binding domains (NBDs) responsible for ATP binding and hydrolysis (Figure 1A). Each half-transporter contains an N-terminal four-transmembrane helix bundle, termed TMD0. A conserved salt bridge between the TMD0 and tapasin located within the ER membrane was found to be essential for PLC assembly and for an efficient antigen processing (8, 9). In contrast, the core TAP subunits lacking these TMD0s are sufficient for TAP assembly, ER targeting, peptide binding, and peptide translocation (8, 10). The core transporter and the TMD0s are connected by elbow helices (EHs), whose function is still undefined. The coupling helices CH1 and CH2, located in the cytosolic loops (CLs) between TM2 and TM3 as well as TM4 and TM5, allow a cross talk between the TMDs and the NBDs *in cis* and *in trans*. They are embedded into a groove between the RecA-like and the α-helical domains of the NBD, thereby interacting with the Q- and X-loop as well as the NBD region, which positions the purine base of ATP (Figure 1B) (11, 12).

Several conserved motifs, such as Walker A, Walker B, ABC signature (C-loop), A-/D-/Q-loop, and H-switch, are characteristic for ABC proteins. The X-loop is an additional conserved region but only present in some ABC exporters, such as TAP. Cysteine-scanning and cross-linking approaches revealed that both coupling helices interact *in trans* with the X-loop of the opposite subunit (TEVD**E**AG and TDVG**E**KG; conserved glutamate in bold). The transport activity was reduced without affecting peptide binding, when the conserved glutamate of the X-loop in TAP2 was mutated. Cross-linking the X-loop with either CH1 or CH2 impedes substrate transport or binding, respectively (11, 12).

The non-equivalence of the two nucleotide-binding sites (NBS I and II), each coordinating an ATP molecule by both NBDs, is an intriguing feature common to many human ABC transporters including TAP (Figure 1C). In TAP1, the conserved glutamate next to Walker B, acting as catalytic base, is replaced by aspartate and the conserved histidine of the H-switch by glutamine. In addition, the signature motif (C-loop) of TAP2 differs by two residues (LSGGQ to LAAGQ). Altered residues are exclusively located at NBS I, which displays a strongly diminished ATPase activity. NBS I is hence qualified as a non-consensus site. The role of this degenerate NBS in ABC transporters is still enigmatic. However, a peptide-specific trapping of an ATP hydrolysis transition state at both NBS can only be observed after one cycle of ATP hydrolysis and not in a backward reaction in the presence of ADP and trapping reagent (15). Nonetheless, a mutated TAP complex harboring two degenerate C-loops shows a drastically diminished transport rate. Thus, NBS I seems to adopt a regulatory role, while the consensus site constitutes the driving motor for substrate transport by TAP. This was supported by an increase in transport activity for a chimera with two canonical C-loops (16, 17).

## Transport Mechanism of Peptides by TAP

**Figure 2:**
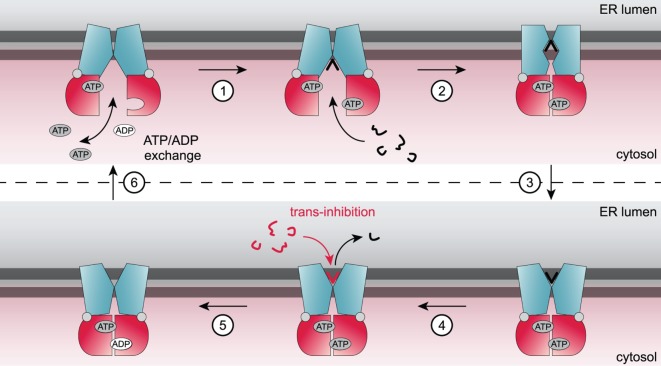
**Current model of the translocation cycle of peptides by transporter associated with antigen processing (TAP)**. Under physiological conditions, TAP is loaded with ATP in an inward-facing conformation. Binding of a peptide (step 1) induces a conformational rearrangement of the TAP complex and subsequently nucleotide-binding domain dimerization. Presumably, an occluded state is formed (step 2) followed by a switch to an outward-facing conformation triggering peptide translocation across the membrane (step 3) and subsequent release of the peptide into the endoplasmic reticulum (ER) lumen (step 4). ATP hydrolysis (step 5) resets the TAP complex back in its resting state and ADP is exchanged against ATP (step 6). At high ER-lumenal peptide concentrations (16 µM), TAP is blocked by trans-inhibition.

Details of the transport mechanism and conformational dynamics of the TAP transporter have not been elucidated adequately to date. The current working model of the translocation mechanism of peptides by TAP was derived from biochemical approaches and recent structures of ABC exporters, which share a similar overall architecture (Figure 2). The TMDs of the TAP complex seal the pathway to the ER lumen in the inward-open conformation. Peptide binding to the TMDs occurs independently from ATP binding to the NBDs, which are separated from each other (15–17). Under physiological conditions, the transport complex is loaded with two ATP-Mg in the resting state. Peptide binding of TAP triggers an allosteric cross talk between NBDs and TMDs transmitted by the coupling helices. The adopted substrate-bound state induces dimerization of the NBDs and presumably the formation of an occluded state. A subsequent conformational rearrangement of the TMDs switches TAP from the inward-facing state to the outward-facing state. The tight NBD dimer sandwiches two ATP molecules at its interface. As discussed above, each ATP molecule is tightly coordinated in the NBS by both NBDs (18–20). Notably, ATP hydrolysis by TAP is strictly coupled to peptide binding and translocation. No basal ATPase activity has been observed in the absence of peptide substrates (12, 21, 22). ATP hydrolysis is coupled to the peptide release into the ER lumen. After ATP hydrolysis, the NBD dimer is destabilized and the transporter resets to its inward-open, “resting” state, in which inorganic phosphate and ADP are exchanged by ATP-Mg (23–25).

## Substrate Binding of Human TAP

Peptide recognition by TAP is the initial step of the translocation cycle followed by allosterically coupled conformational changes and ATP hydrolysis. Kinetic analysis of peptide binding revealed a two-step process consisting of a fast association and a slow conformational rearrangement of the transporter (26). The peptide-binding process is characterized by a high activation energy and involves 25% of all TAP residues (27). Peptide and ATP binding occur independently from one another, but peptide binding and translocation induce ATP hydrolysis (21). Fluorescence cross-correlation spectroscopy revealed that only one peptide at a time can bind per TAP complex (22). TAP is able to bind peptides consisting of 8–16 amino acids with similar nanomolar affinities (16). Furthermore, binding of peptides with up to 40 amino acids or bulky side chains, such as fluorophores, spin probes, chemical proteases, or polylysine chains, is not impeded (21, 28–30). For peptide selection by TAP, the first three N-terminal residues and the C-terminal peptide residue are critical (17, 28, 29, 31–35). The recognition principle of human TAP was investigated by applying combinatorial peptide libraries including one defined residue in a scanning approach, whereas all other positions are fully randomized (36). These studies revealed that human TAP favors positively charged (positions 1 and 2) as well as aromatic residues (position 3) at the N-terminal positions and hydrophobic or basic amino acids at the C terminus of the peptide. Free N and C termini are an important prerequisite for high-affinity binding (15, 36). The region between these N- and C-terminal “anchor” residues can largely vary in sequence and length. EPR spectroscopy provided first insights into the dynamics and structure of TAP-bound peptides (29). The anchor residues are restricted in motion, while residues in-between are highly flexible. Notably, the distance between the N and C termini of the TAP-bound peptide was determined to approximately 2.5 nm by double electron–electron resonance experiments independently of peptide length (29). These data suggest that longer peptides accommodate an extended kinked structure in the TAP-bound state.

**Figure 3:**
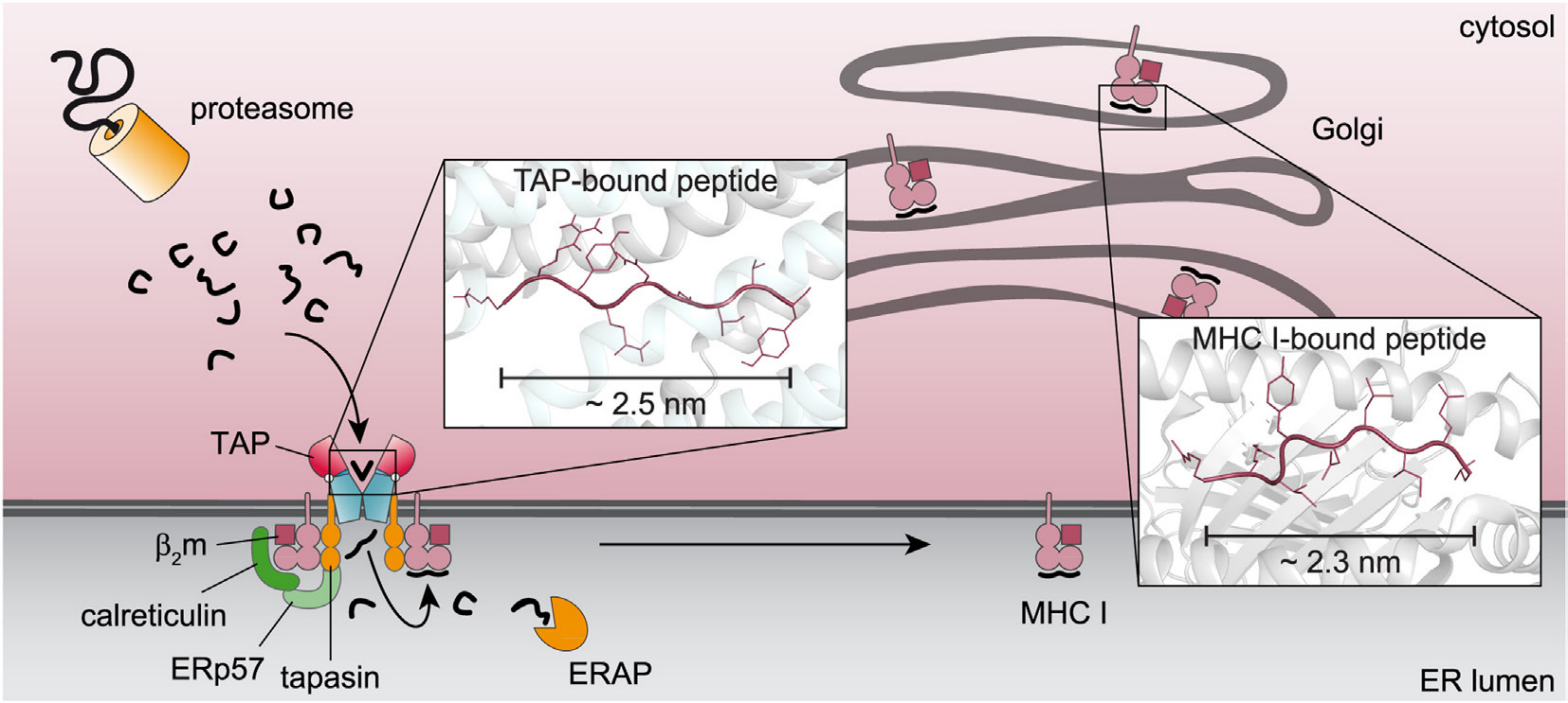
**Coevolution of key machineries in the pathway of major histocompatibility complexes (MHC) I antigen processing**. Proteasomal degradation products with a preferential length and a hydrophobic C terminus are recognized by the transporter associated with antigen processing (TAP) complex and translocated into the endoplasmic reticulum (ER) lumen. Peptides, which do not fit into the MHC I binding pocket, are N-terminally trimmed by the ER-resident aminopeptidase (ERAP) and subsequently loaded onto MHC I molecules for further processing. The similar C-terminal anchor residues and the overlapping N-to-C distance of TAP- and MHC I-bound peptides [(13), PDB: 2BSR] point to a coevolution of both components in antigen processing.

The structurally defined N-to-C distance of TAP-bound peptides and the recognition principles indicate a coevolution of the immunoproteasome, TAP, and MHC I to improve antigen presentation (Figure 3). The immunoproteasome, whose assembly is stimulated by interferon-γ, preferably generates peptides equipped with hydrophobic or basic C termini, which are favored by TAP (37). The TAP complex translocates these peptides into the ER lumen. Longer peptides are N-terminally trimmed by the ER-resident aminopeptidase (ERAP) to fragments containing mostly eight or nine amino acids. In addition to the favored C-terminal residue, these peptides preferentially fit into the MHC I binding pocket (38). X-ray crystal structures have shown a fixed N-to-C distance of MHC I-bound peptides, defining the minimal length of MHC I ligands (39). Thereby, N and C termini of the peptide bind to the A and F pockets of the MHC I binding groove. A predominant N-terminal anchor residue is located at position 2, whereas a second hydrophobic anchor residue rests at the C terminus of the peptide (1, 39). T-cell receptors conversely recognize peptide residues at positions located between the anchor residues of the TAP transporter (40).

Besides the well-characterized high-affinity peptide-binding site accessible in the inward-facing conformation, a second, low-affinity binding site has been proposed based on transport studies of TAP reconstituted in proteoliposomes (41). Translocation of peptides into proteoliposomes did not exceed a lumenal peptide concentration of about 16 µM, although TAP is an active, unidirectional transporter. This maximal peptide concentration is independent from the number of TAP complexes in the membrane. Thus, an inhibition *in trans* points to a second low-affinity binding site facing the ER lumen (Figure 2). Saturation of the ER-lumenal peptide-binding site is suggested to impede the transporter from switching back to the inward-facing conformation and inducing another transport cycle. This process, also called trans-inhibition, might prevent the induction of ER stress and an unfolded protein response at high peptide concentration in the ER lumen (41).

## Substrate-Binding Site of TAP

**Figure 4:**
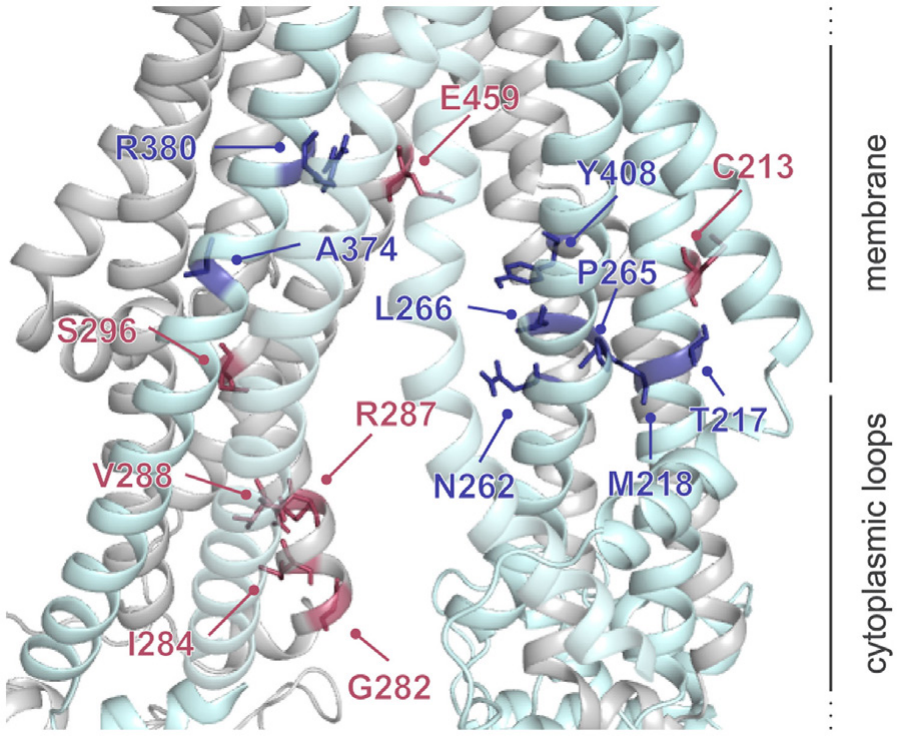
**Residues involved in peptide binding and translocation**. Cross-linking studies (raspberry) and mutational analyses (blue) revealed transporter associated with antigen processing (TAP) residues contributing to substrate-binding/translocation (see Table 1). The 3D homology model of the human TAP complex is based on the TAP homolog TmrAB in the inward-facing conformation (13, 14).

A number of residues and sequence regions in human TAP are critical for peptide binding and transport (Figure 4; Table 1). Initial photo-cross-linking studies mapped the peptide-binding site within the TMDs of the coreTAP complex, which are confined to the CLs between TMH4 and TMH5 (P375-M420^TAP1^ and R354-M389^TAP2^) as well as the linker region between the TMD and NBD of each half-transporter (Q453-R487^TAP1^ and I414-M433^TAP2^) (42). In addition, residues G282/I284/R287/V288 within the CL1 of TAP1 were identified as a peptide sensor region involved in inter-domain cross talk to allosterically couple peptide binding and ATP hydrolysis (28). Furthermore, several residues important for substrate specificity were determined. The deletion or substitution of E263^TAP1^ in murine or human cells caused a phenotype comparable to TAP1-deficient cells as shown by MHC I surface expression. In this TAP mutant, peptide binding and translocation are impaired (43). A374 and C213 of TAP2 were identified to control the peptide repertoire (42, 44). The replacement of C213^TAP2^ by serine leads to an altered substrate specificity of human TAP. In this mutant, negatively charged peptide residues are favored at positions 1, 2, and especially at the C terminus, as determined by a scanning approach using combinatorial peptide libraries. Cross-linking approaches uncovered the C-terminal peptide residues directly contacting C213^TAP2^ (44). Surprisingly, this finding is contradictory to homology models of the TAP transporter, in which C213^TAP2^ points toward the membrane bilayer (12, 13, 45, 46). Cross-linking studies and mutational analysis on rat TAP derived additional residues within the TAP complex, which were suggested to control substrate specificity or to contribute to the peptide-binding site. In addition, several other residues in TAP2 (A217, E218, Q380, Q262, S265, and L266 in rat, corresponding to T217, M218, R380, N262, P265, and L266 in human) were identified to modulate the peptide specificity (30, 47). Recently, residues C273, Y385, and E436 were determined in rat TAP1 (corresponding to S296, Y408, and E459 in human TAP) to directly coordinate the bound peptide (46).

**Table 1:** **Essential residues for the functionality of transporter associated with antigen processing (TAP)**.

	TAP1	TAP2	Putative function	Reference
Peptide sensor	G282^XL^, I284^XL^, R287^XL^, V288^XL^	–	Peptide sensing	(28)
Binding region	P375-M420^XL^, Q453-R487^XL^	R354-M389^XL^, I414-M433^XL^	Peptide binding	(42)
Substrate specificity	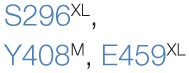	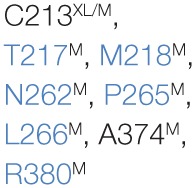	Altering of epitope repertoire	(30, 42, 44, 46, 47)
Functionality	E263^M^	–	Impaired binding/transport	(43)

*^XL^/^M^residues of human (black) and rat (blue) TAP derived from cross-linking and mutagenesis studies, respectively; all residue numbers refer to human TAP*.

Despite the identification of residues presumably contributing to the substrate-binding site, evaluation of the location of the substrate-binding pocket of the TAP complex and detection of a peptide epitope within this pocket are challenging. Molecular docking approaches were applied to enlighten this aspect. The first docking study of the HLA-B27 epitope RRYQKSTEL was based on a TAP homology model derived from the homodimeric ABC transporter ABCB10 (45). Electrostatic interactions between charged peptide residues and the binding pocket itself are likewise required for peptide binding, in addition to free N and C termini of the peptide. Calculations of electrostatic potentials of the predicted binding site indicated the presence of two binding pockets. One binding pocket is negatively charged and binds the peptide N terminus, while the other is positively charged and coordinates the C terminus of the TAP-bound peptide. Here, a bound nonamer adopts an extended conformation parallel to the membrane plane with an N-to-C distance of 2.2 nm, in line with pulsed EPR distance measurements (29). These docking studies restrained the TAP interaction sites to the elucidated electrostatic binding pockets, while the peptide dynamics was not restricted. A second docking study combined recently identified residues with a TAP homology model based on the homodimeric ABC transporter Atm1 (46). This study proposed that peptides bind to TAP in a β-hairpin-like conformation parallel to the membrane plane (46), which is contrary to EPR distance constraints on TAP-bound peptides (29) and docking studies (45). Even though docking of a double spin-labeled β-hairpin peptide resulted in restricted spin labels within the TAP-binding site with an inter-spin distance of ~2.3 nm (46), this peptide conformation does not explain the restriction of TAP-binding peptides to a minimal length of eight residues. Therefore, the peptide conformation in the TAP-bound state was analyzed by solid-state NMR to clarify these controversial results.

**Figure 5:**
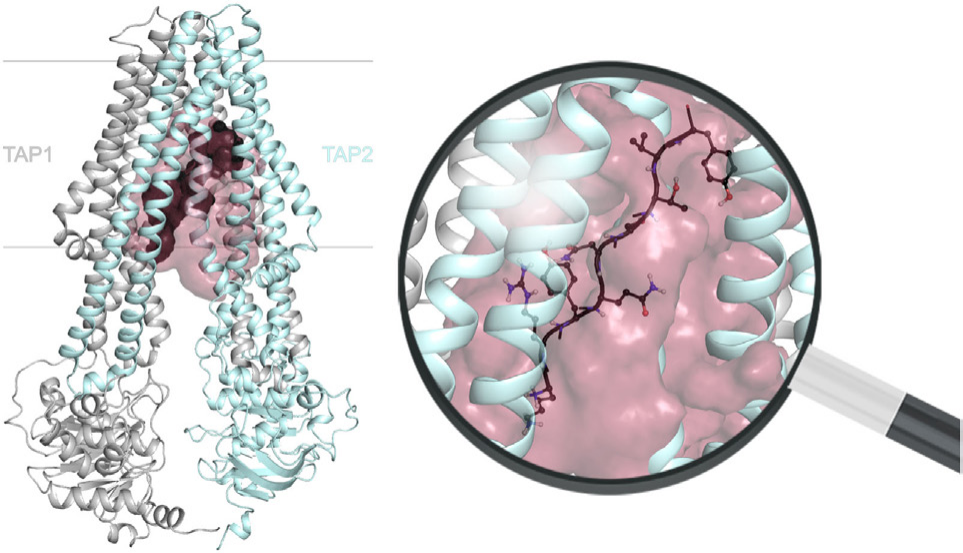
**Location of the substrate-binding site within the transporter associated with antigen processing (TAP) complex**. The transmembrane domains position the peptide (dark gray) *via* its N and C termini between TAP1 (gray) and TAP2 (cyan). The extended peptide backbone orients in a tilted position with respect to the membrane plane and is surrounded by a large cavity (raspberry). The magnification of the substrate-binding site illustrates a bound nonamer in a ball-and-stick representation (red: O, blue: N, white: H), whereas side chains are only shown for one conformer. The 3D homology model of the TAP complex is premised on the heterodimeric ABC-binding cassette transporter TmrAB in the inward-facing conformation (13, 14).

Dynamic nuclear polarization-enhanced solid-state NMR enabled the elucidation of an extended backbone conformation of TAP-bound peptides at an atomic resolution due to a large signal enhancement and to significantly reduced data acquisition times (13). Molecular docking of the peptide with a restricted backbone but freely rotating side chains into a TAP homology model based on the heterodimeric ABC transport complex TmrAB revealed the peptide bound to TAP in a tilted orientation with respect to the membrane plane (Figure 5). Most of the peptide coordinating residues of TAP coincided with residues identified by biochemical methods in previous studies. The obtained backbone structure further supports the coevolution of TAP and MHC I molecules already discussed above (Figure 3). Chemical shift analyses provided new insights into the peptide–TAP interaction. The peptide is coordinated by TAP at positions 1, 3, and 9. The study further unveiled a flexibility of the peptide within its binding site, since two distinct binding modes were observed for the N-terminal amino group. Interestingly, a large cavity formed by the TMDs of both half-transporters can be found next to the binding sites of the peptide anchor residues. This cavity may allow accommodation of bulky side chains of the peptide and even covalently attached fluorophores (Figure 5).

## Concluding Remarks

Several biochemical and theoretical attempts to localize the substrate-binding site in the TAP complex now result in a better picture of substrate selection by the transporter. Although molecular docking approaches provided a useful discernment of potential locations for substrate-binding sites, these assays are biased by restraining the conformational freedom of the peptide and the potential number of hydrogen donors and acceptors. The recently elucidated backbone structure and the precise distance measurements of the N and C termini of TAP-bound peptides provided experimental evidence that significantly improved these molecular docking approaches. However, further refinements of the positions of the binding pocket(s) by, e.g., pulsed EPR spectroscopy are required. These studies will be complemented with high resolution X-ray crystallography analyses of peptide–TAP complexes. Alternatively, cryo-EM structures may also provide valuable insights into the substrate-binding region as demonstrated for the TAP-related ABC transporter TmrAB (48) and the TAP transporter with bound viral inhibitor ICP47 (49).

Unprecedented insights into the dynamics of peptide binding and translocation by TAP will be addressed by applying advanced biophysical techniques, such as single-molecule Förster resonance energy transfer. Together with the depicted peptide-binding site, these studies will significantly boost the overall understanding of substrate translocation by TAP and thus provide the basis to develop novel drugs or therapeutic approaches. Despite the wealth of biochemical data on the high-affinity substrate-binding site, thermodynamic and kinetic characterization of the low-affinity binding site is incomplete to date. The substrate specificity of the latter site and its location within the TAP complex will be of great interest in prospective studies. These investigations on the TAP complex will also be pioneering for other ABC transporters, such as TAP-like (ABCB9) (50), revealing similar trans-inhibitory effects. Moreover, it will be decisive to determine how trans-inhibition of TAP helps to balance antigen processing as well as ER homeostasis and how these processes are involved in the control of ER stress caused by accumulated peptides.

## Author Contributions

EL prepared the figures and tables. EL and RT wrote the manuscript.

## Conflict of Interest Statement

The authors declare that the research was conducted in the absence of any commercial or financial relationships that could be construed as a potential conflict of interest.
